# COVID‐19 and the pulmonary complications of sickle cell disease

**DOI:** 10.1002/jha2.105

**Published:** 2020-10-08

**Authors:** Thivya Sivalingam, Baba Inusa, Pat Doyle, Eugene Oteng‐Ntim

**Affiliations:** ^1^ Faculty of Life Sciences and Medicine GKT School of Medicine King's College London London UK; ^2^ Department of Paediatrics Evelina Children's Hospital, Guy's and St Thomas' Hospital NHS Foundation Trust London UK; ^3^ Department of Non‐communicable Disease Epidemiology London School of Hygiene and Tropical Medicine Faculty of Epidemiology and Population Health London UK; ^4^ Department of Obstetrics and Gynaecology Guy's and St Thomas' Hospital NHS Foundation Trust London UK

**Keywords:** infection, sickle cell anemia, sickle cell disease, therapy, viruses

## Abstract

Sickle cell disease (SCD) patients are commonly affected by pulmonary complications such as acute chest syndrome (ACS), pulmonary embolism (PE) and pneumonia that contribute to significant mortality risks. With a greater susceptibility to infection, they are deemed to be vulnerable patients during the current COVID‐19 pandemic. In emerging small case studies of SCD patients with COVID‐19 and further complicated by pneumonia, ACS, and/or PE, the clinical benefits of early exchange transfusion and Tocilizumab are evident. However, further clinical trials and larger cohort studies are essential to evaluate effective diagnostic and management options for this high‐risk group.

## 1 INTRODUCTION

It is significant to address the impact of the current COVID‐19 pandemic on sickle cell disease (SCD) patients who are particularly vulnerable. This novel severe acute respiratory syndrome coronavirus 2 (SARS‐CoV‐2) strain causes pneumonia of variable severity, with severe illness in approximately 15‐20% of infected patients. The main clinical presentations can include a persistent dry cough, fever (≥37.3°C) and dyspnea [1]. However, in many cases no signs or symptoms are present, rendering the situation more challenging to control, particularly as a droplet infection.

The United Kingdom has reported the most deaths in Europe with a higher risk of mortality in those of Black, Asian, and Minority Ethnic (BAME) groups. In particular, Black Afro‐Caribbean individuals had a 1.9‐fold increased risk in mortality compared to Caucasians [2]. Similar statistics were also observed in many South Asian ethnic groups. SCD patients are predominantly of BAME background and are more susceptible to infections due to manifestations such as functional asplenia and impaired complement activation [3]. Therefore, this patient population is potentially at elevated risks of morbidity and mortality.

There is limited evidence on the impact of the previous SARS‐COV viral pandemic on SCD patients. However, a retrospective cohort study on SCD patients during the H1N1 viral pandemic in 2009 revealed that 34% of patients with the viral H1N1 infection developed acute chest syndrome (ACS), while 13% of those with the seasonal influenza developed ACS [4]. Such patients more commonly required exchange transfusion and mechanical ventilation, indicating the presence of severe ACS episodes. It is likely that COVID‐19 induces a similar if not a more significant impact on the pulmonary conditions of SCD.

## 2 OVERLAP OF COVID‐19 AND SCD‐RELATED PULMONARY COMPLICATIONS

An emerging number of case studies have suggested important associations between ACS and COVID‐19. ACS is a leading cause of intensive care admissions and mortality in SCD patients [5]. It is a complex pulmonary condition often triggered by an atypical bacteria or less commonly by a virus. ACS and pneumonia can commonly be misdiagnosed due to comparable clinical presentations such as a fever, cough, dyspnea, and chest pain. Consequently, a delay in appropriate management due to challenges in the true diagnosis may lead to worsening of the patient's condition. Further to mimicking the symptoms, evidence suggest that SARS‐COV‐2 infection may also provoke a severe clinical course of ACS [6, 7].

Beerkens et al reported the case of a 21‐year old patient with the HbSβ⁰‐thalassemia SCD subtype who had developed ACS as a result of COVID‐19 [6]. It emphasizes the need for much vigilance even in younger adults, particularly immunocompromised individuals. Moreover, patients from two case studies had been admitted primarily for a vaso‐occlusive crisis (VOC) characterized by minor to severe pain with no initial presentations of flu‐like symptoms [6, 7]. The suspicion of a COVID‐19 infection in the following days, due to a new onset of fever, increase in dyspnea, pain, deteriorating oxygen saturation levels, and abnormalities in chest radiographic imaging, was confirmed using the reverse transcription polymerase chain reaction (RT‐PCR) test. This suggests that COVID‐19 may precede and hence trigger ACS without any initial clinical manifestations of the viral infection. Therefore, SCD patients admitted for a suspected ACS episode may benefit from an early RT‐PCR test for SARS‐COV‐2 even if signs and symptoms of an infection are absent.

In addition, recent findings from a retrospective cohort study reveal that approximately 23% of patients with severe COVID‐19 also had an acute pulmonary embolism (PE), as identified on computed tomography pulmonary angiogram (CTPA) [8]. This is particularly deleterious to SCD patients who have a 3.5‐fold prevalence of pulmonary emboli as a result of their hypercoagulable state [9]. Hence, SCD patients with COVID‐19 may be susceptible to even greater risks of developing PE. This further underlines the need for additional diagnostic measures such as chest X‐rays and CTPA to help identify pulmonary complications that are more likely to accompany COVID‐19 induced pneumonia. Chest radiography displaying a new pulmonary infiltrate is important in diagnosing ACS [5]. On the contrary, positive cases of COVID‐19 may not always display its signs on a chest X‐ray, as highlighted in Patient 2 in the case study by Nur et al [7]. Therefore, using a more sensitive imaging method such as chest CT scans can help identify COVID‐19 signs missed on chest X‐rays.

## 3 MANAGING SCD PATIENTS INFECTED WITH COVID‐19

There is an obvious risk to sickle cell patients during this pandemic, hence such individuals should be closely monitored if presenting with any form of respiratory complications and should have a lower threshold for initiating treatment. Medical histories of SCD patients are essential in guiding management choices, as those with a previous history of ACS events are more likely to develop future episodes. A number of studies have proposed the benefits of using Tocilizumab (TCZ), an anti‐interleukin 6 (IL‐6) receptor monoclonal antibody, in critical SCD patients with COVID‐19.

To exemplify, Odièvre et al reported on the significant improvement in the severe respiratory status of a 16‐year‐old SCD patient (HbSS genotype), who developed ACS after the COVID‐19 infection and was further complicated by bilateral pulmonary embolism [10]. Four days after initiating one pulse of intravenous TCZ, she required no additional respiratory support and thereafter discharged from the intensive care unit. IL‐6 can be found at particularly elevated levels in the normal state of SCD patients but is further increased during VOC [11]. SARS‐COV‐2 proteins have also demonstrated to induce the upregulation of proinflammatory cytokines including IL‐6 [12]. Such small case studies highlight the importance of addressing cytokine release syndrome for the management of severe clinical scenarios of SCD patients with COVID‐19 and further comorbidities like ACS and/or PE.

Careful early management is vital to prevent progression to the life‐threatening complication of acute respiratory distress syndrome (ARDS). In addition to the standard therapy such as fluids, oxygen, and broad‐spectrum‐antibiotics to treat suspected ACS in COVID‐19 patients, it may be important to consider early exchange transfusion and/or hydroxyurea therapy that can prevent and diminish red blood cell (RBC) sickling [13]. Beerkens et al supports this pre‐emptive consideration of early exchange transfusion in patients with ACS and COVID‐19, having observed the clinical and respiratory improvement from its use in a critically ill patient after other forms of therapy had failed [6].

On the contrary, a cohort study of 83 SCD inpatients with COVID‐19 at a French center suggested that neither transfusions nor hydroxyurea treatment were significant in preventing ICU admissions in a small subgroup [14]. The study results also inferred that SCD patients, typically a young population, may be at reduced risks of morbidity and mortality as increased disease severity is more commonly seen in those older than 45 years. In fact, a recent large observational study of 725 COVID‐19 positive patients highlighted that only nine of 725 had SCD. Moreover, in comparison to their aged match controls the SCD group required a significantly lower number of ICU admissions and had no mortalities, despite their immunocompromised state [15]. This raises the question whether SCD (HbSS) may have protective effects against a severe COVID‐19 infection, and if so, whether they are due to better healthcare surveillance of SCD patients or due to factors at a molecular level. Such conflicting evidence will require investigations at a larger scale to establish a more significant conclusion regarding the mortality and morbidity risks, and the prophylactic management of SCD patients with COVID‐19 if necessary.

## 4 SUMMARY

Overall, sickle cell patients are an important patient cohort who require much vigilance during the current COVID‐19 pandemic. As a vulnerable group, government recommendations had emphasized sickle cell anemic patients to self‐isolate for much longer (12 weeks) than the general population. However, prolonged periods of lockdown and the consequent lack of physical activity can increase the risks of complications. Furthermore, there are challenges in providing regular treatment for sickle cell patients, for example, since the pandemic a decline in blood donors due to an increased risk of infection transmission is expected.

Moreover, in low‐resource countries particularly in rural areas and refugee camps, the lack of technical equipment and treatment availability can render it challenging to distinguish and manage the pulmonary complications in SCD patients during this pandemic. As infection rates of COVID‐19 in Africa and India have accelerated, it is significant to evaluate appropriate diagnostic and management options as such countries account for the main geographical locations concentrated with the indigenous SCD population. Globally, large cohort studies of SCD patients with COVID‐19 is profoundly lacking, particularly in developing countries. More research is essential in such a high‐risk population to recommend optimal management options and to better understand patterns of COVID‐19 and SCD severity in patients of different ages and SCD genotypes.

## CONFLICT OF INTEREST

The authors declare no conflict of interest.
